# Case Report: Anti-ADAM23 antibody: an overlooked autoantibody against VGKC-complex in autoimmune encephalitis

**DOI:** 10.3389/fimmu.2025.1589360

**Published:** 2025-05-30

**Authors:** Pinfei Ni, Lin Bai, Nan Jiang, Siyuan Fan, Ming Yao, Haitao Ren, Hongzhi Guan

**Affiliations:** Department of Neurology, Peking Union Medical College Hospital, Chinese Academy of Medical Sciences and Peking Union Medical College, Beijing, China

**Keywords:** autoimmune encephalitis, voltage-gated potassium channel complex, anti-ADAM23 antibody, tissue-based assay, cell-based assay

## Abstract

**Objectives:**

Antibodies against voltage-gated potassium channel (VGKC) complexes include anti-LGI1 and anti-CASPR2. Anti-ADAM23 antibodies have not been previously reported in autoimmune encephalitis (AE).

**Case report:**

We report a 71-year-old female patient who presented with rapidly progressive short-term memory loss, psychobehavioural abnormalities, and impaired consciousness. Six months earlier, she had recovered from herpes simplex encephalitis (HSE). Cerebrospinal fluid (CSF) analysis suggested lymphocytic inflammation. AE was considered after the exclusion of HSV reactivation and other infectious etiologies. A comprehensive screening for commercially available autoimmune and paraneoplastic antibodies yielded negative results. A tissue-based assay (TBA) of CSF revealed a pattern of neuropil reactivity (neuropil pattern, suggesting the presence of neuronal surface antibodies (NS-Ab). A laboratory-developed antibody panel screening using a cell-based assay (CBA) identified an anti-ADAM23 antibody. Immunofluorescence staining showed that ADAM23 colocalized with Tuj1, which is expressed on the surface of neurons. Subclass analysis revealed that the anti-ADAM23 antibody predominantly belonged to the IgG4 subclass. Finally, the patient was diagnosed with an anti-ADAM23 antibody-associated AE. During the early stages of the disease, the patient underwent several courses of intravenous immunoglobulin (IVIg) with limited efficacy. Following the addition of high-dose methylprednisolone pulse therapy, the patient's consciousness improved from coma to stupor and the seizures were relieved.

**Conclusion:**

We propose that anti-ADAM23 antibody, a previously overlooked autoantibody targeting the VGKC complex, may have diagnostic significance for autoimmune encephalitis.

## Introduction

In 2010, leucine-rich glioma inactivated 1 (LGI1) and contactin-associated protein 2 (CASPR2) were identified as the major target antigens in patients with positive voltage-gated potassium channel (VGKC) complex antibodies detected by radioimmunoassay using cell-based assays (CBAs) (1). Anti-VGKC-complex-positive but anti-LGI1/CASPR2 negative patients may present with autoimmune encephalitis (AE), also reported in several non-autoimmune diseases, including Creutzfeldt–Jakob disease and multiple system atrophy with limited response to immunotherapy and poor correlation between clinical data and serial antibody levels (2). These findings emphasize the importance of defining these disorders according to the molecular identity of the targets (LGI1 or CASPR2) and caution against the use of VGKC complex antibodies for the diagnosis and treatment of patients without further definition of the antigen (3). However, a large serological study further revealed that many patients with high titers of VGKC-complex antibodies lack specificity for LGI1 and CASPR2, suggesting that other antigen components within the VGKC-complex remain to be identified (4). We report a case of autoimmune encephalitis that occurred 6 months after the resolution of HSE. The combination of TBA and CBA identified a novel autoantibody against the VGKC-complex:anti-ADAM23 antibody.

## Case description

**Figure 1:**
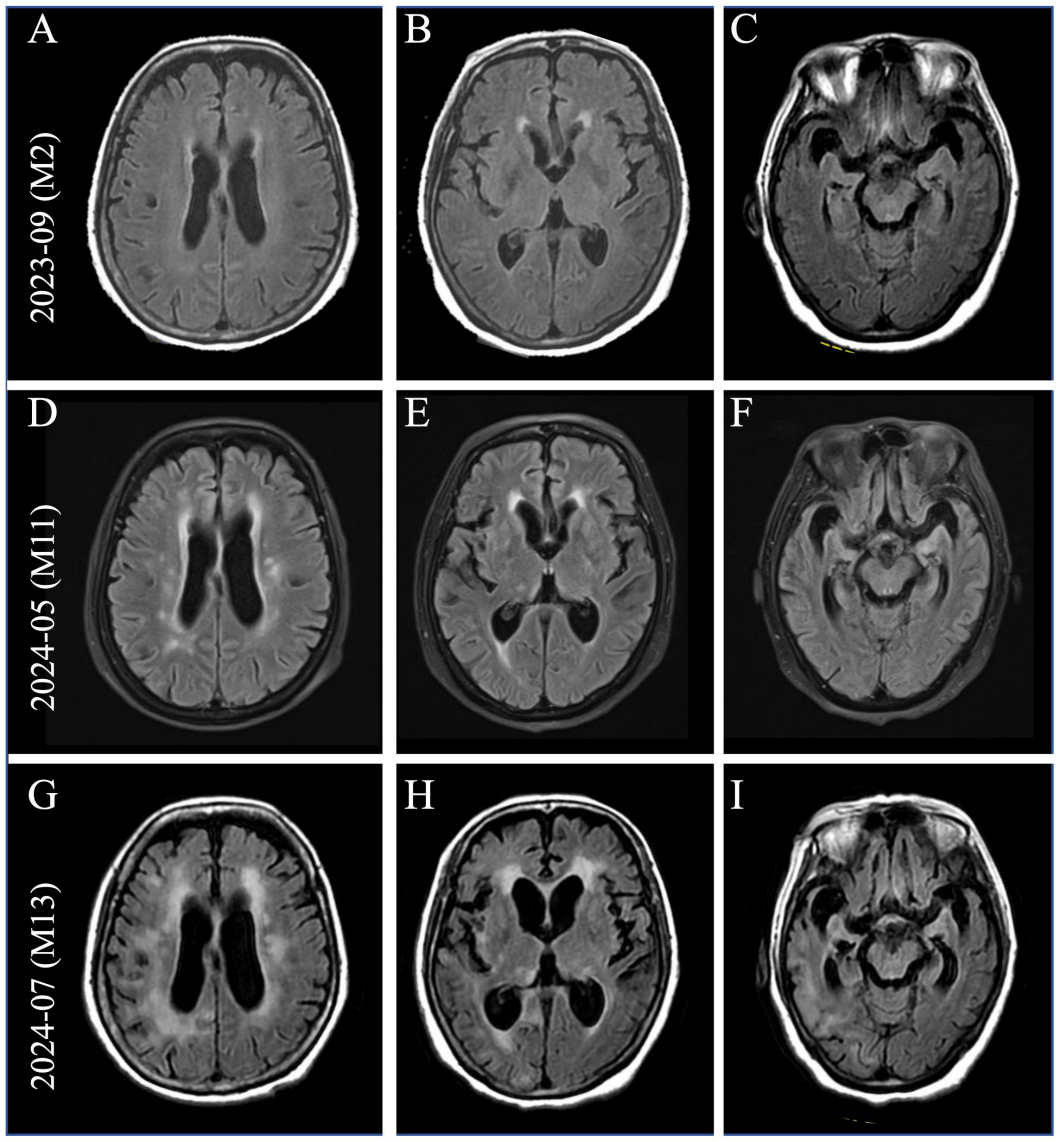
Brain imaging at the time of clinical progression. **(A–C)** Brain MRI (pictured: axial T2 fluid-attenuated inversion recovery) showed non-specific white matter changes two months after onset. With the development of severe neurological symptoms, brain MRI showed gradually increasing white matter hyperintensities (WMHs) in the periventricular regions **(D, G)**, bilateral basal ganglia **(E, H)**, and progressive brain atrophy **(F, I)**.

In July 2023, a 71-year-old woman presented with fever, headache, psychobehavioral abnormalities, and disorder of consciousness. Brain MRI revealed non-specific white matter changes (**Figures 1A–C**). CSF evaluation revealed pleocytosis (96 × 10^6^/L, 90% lymphocytes) with elevated protein (1.52 g/L). Empirical acyclovir treatment (10 mg/kg, q8h) was immediately initiated for suspected viral encephalitis. Serum and CSF tested negative for commercial neuronal autoantibodies. Subsequent next-generation sequencing (NGS) identified herpes simplex virus type 1 (HSV-1), confirming the diagnosis of HSE. Given that the patient had severe HSE and comorbid systemic infection, IV immunoglobulins (2 g/kg, 5 days) were added for immunomodulation. She was discharged in October 2023 with an excellent response.

**Figure 2:**
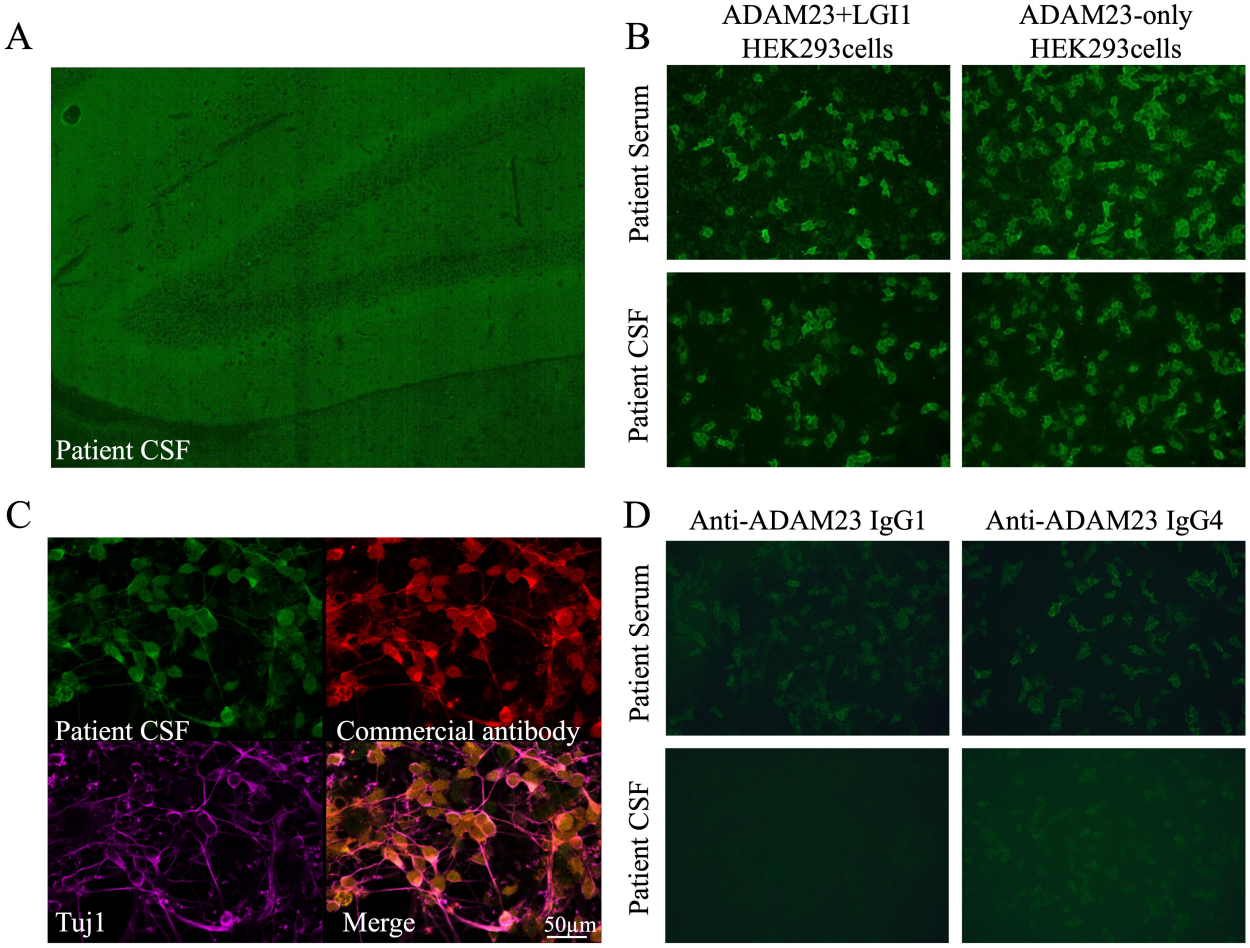
Identification of unknown fluorescent objects in the rat hippocampus. **(A)** Tissue-based assay showing the binding of IgG from the patient’s CSF to the molecular layer of the dentate hilus and dentate gyrus in the rat hippocampus. **(B)** HEK293 cells expressing ADAM23 + LGI1 and ADAM23-only were positive in the patient’s serum and CSF. **(C)** Immunofluorescence staining showed anti-ADAM23 antibody in the patient’s CSF, consistent with the commercial anti-ADAM23 monoclonal antibody, co-localized with the neuronal cytoskeletal protein Tuj1. **(D)** Autoantibodies include subclasses of IgG1 and IgG4, predominantly IgG4.

In April 2024, she rapidly developed progressive short-term memory loss, psychobehavioral abnormalities, and a disorder of consciousness. Brain MRI showed white matter hyperintensities (WMHs), particularly in the bilateral basal ganglia, thalami, and periventricular regions (**Figures 1D–F**). CSF evaluation showed pleocytosis (10 × 10^6^/L, 80% lymphocytes), with elevated protein (0.96 g/L) and interleukin-6 (IL-6; 631 pg/mL, reference range <5.9) levels. Infectious screening of the CSF was negative. Commercial neuronal autoantibodies (including anti-NMDAR, LGI1, GABA_B_R, CASPR2, GAD65, AMPAR, DPPX, and IgLON5) and paraneoplastic antibodies (including anti-Ho, Yo, Ri, Amphiphysin, CV2/CRMP5, PNMA2) were negative in the serum and CSF. However, the TBA of CSF in the hippocampus showed that the molecular layer of the dentate hilus and dentate gyrus was stained, which constitutes a “neuropil pattern” (**Figure 2A**). This finding led to suspicion of AE. IV immunoglobulins (2 g/kg, 5 days) were initiated; however, the clinical improvement was not significant. In June 2024, she developed frequent focal seizures and declined consciousness to a comatose state with a CSF IL-6 level >1,000 pg/mL. Due to the unclear diagnosis and side effects of high-dose methylprednisolone pulse therapy, she received IV methylprednisolone (80 mg daily for 14 days, followed by oral prednisone 1 mg/kg daily). One months later, CSF evaluation showed pleocytosis (10 × 10^6^/L, 83% lymphocytes), with mild elevated protein (0.52 g/L) and IL-6 (18 pg/mL).

She was referred to our center for a second opinion in July 2024 because of a progressive disorder of consciousness. The patient had no family history of psychiatric or cognitive disorders. Physical examination of the neurological system revealed coma, with a Glasgow Coma Scale (GCS) score of E1V2M3, and bilateral Babinski signs were positive. Brain MRI showed that brain atrophy was more obvious than before (**Figures 1G–I**). CSF evaluation showed pleocytosis (12 × 10^6^/L, 80% lymphocytes) with re-elevated IL-6 level (669 pg/mL) and some CSF-specific oligoclonal bands. Although the CSF 14-3–3 protein result was positive, the negative outcome of subsequent real-time quaking-induced conversion (RT-QuIC) assays on the CSF ruled out Creutzfeldt–Jakob disease.

To elucidate the unknown fluorescent object observed in TBA, a laboratory-developed antibody panel (including GABA_A_R, mGluR5, ADAM23 + LGI1, Neurexin-3α, and AK5) was employed (**Supplementary Method 1**). Co-transfection of ADAM23 and LGI1 in HEK293 cells was positive in the patient’s serum and CSF, and notably, it was specific to ADAM23, not LGI1 (**Figure 2B**). Immunofluorescence staining showed that the anti-ADAM23 antibody in the patient’s CSF closely overlapped with that of a commercial monoclonal antibody (**Figure 2C**). Co-localization with the neuronal cytoskeletal protein Tuj1 confirmed that ADAM23 was primarily localized to the neuronal cell membrane (**Figure 2C**). Additionally, subclass analysis revealed that the anti-ADAM23 antibody was predominantly present in the IgG4 subclass (**Figure 2D**). To confirm the independent existence of anti-ADAM23 antibody, nine serum and six CSF samples from previously diagnosed anti-LGI1 encephalitis were tested using ADAM23 + LGI1 co-transfected and ADAM23-only transfected HEK293 cells, respectively. None of the samples tested positive for anti-ADAM23 antibodies (**Supplementary Table 1**).

**Figure 3:**
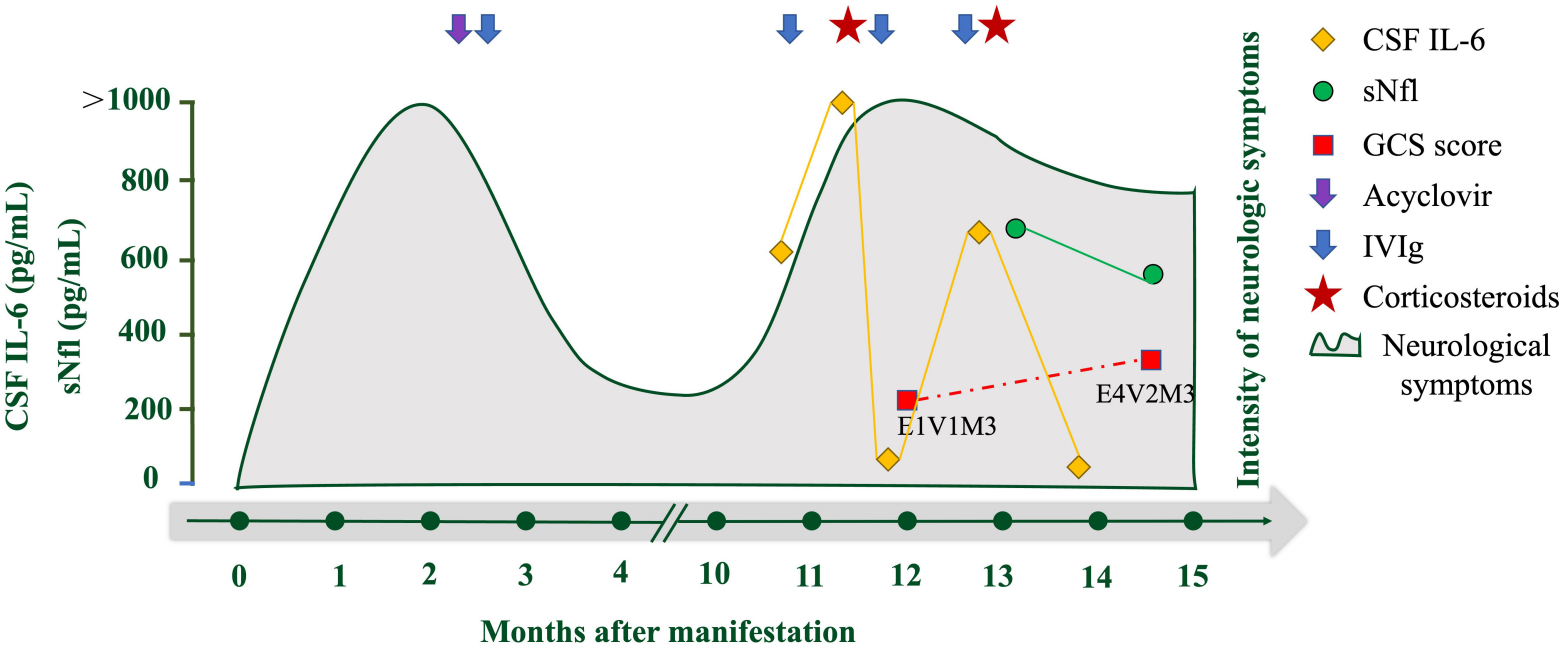
Overview of laboratory parameters and treatment during the clinical course of the disease. Overview of CSF IL-6 (pg/mL; orange rhombus), serum neurofilament light concentration (sNfL, pg/mL; green circle), and GCS score (red square). The purple arrow indicates the application of acyclovir and the blue arrows indicate the application of IV immunoglobulins (IVIG). Red pentagrams indicate corticosteroid use.

The patient was diagnosed with anti-ADAM23 antibody-associated AE. The treatment included IV methylprednisolone (500 mg/day for 5 days, tapered to 80 mg/day for 2 weeks, followed by oral prednisone at 1 mg/kg daily) and IVIg (2 g/kg, 5 days). One month after treatment, her consciousness improved from coma to stupor (E4V2M3), seizures were relieved, and CSF IL-6 levels decreased significantly (15.9 pg/mL). Serum neurofilament light (sNfL), a neuroaxonal damage biomarker, was markedly elevated at disease peak (641.52 pg/mL) but decreased to 564.34 pg/mL post-treatment, correlating with clinical improvement (**Figure 3**).

## Discussion

In this study, we identified a novel autoantibody targeting ADAM23 in a patient with post-herpes simplex encephalitis autoimmune encephalitis (post-HSE AE). The tissue-based assay (TBA) showed a “neuropil pattern,” suggesting the presence of neuronal surface antibodies (NS-Ab). Subsequent screening using a custom antibody panel confirmed the presence of anti-ADAM23 antibodies in the patient’s serum and CSF, with IgG4 being the predominant subclass. A retrospective analysis of nine serum and six CSF samples from patients with anti-LGI1 antibody-associated autoimmune encephalitis found no positivity for anti-ADAM23 antibodies, supporting its independent existence. Clinical improvement following immunotherapy, along with a concurrent reduction in antibody titers, further highlight the diagnostic and potentially pathogenic significance of anti-ADAM23 antibodies.

Approximately 27% of HSE patients develop secondary autoimmune encephalitis (post-HSE AE) (5, 6). Anti-NMDAR is the most common autoantibody in post-HSE AE, accounting for approximately 60% of cases, while about 30% involve unidentified antibodies (6). It has been postulated that the autoimmune response is initiated by antigens released by HSV destruction of neurons, breaking immune tolerance and triggering autoantibody production against the central nervous system (CNS) (7).

TBA is a widely used method for screening autoantibodies in patients suspected of having AE, especially when commercial antibody panels yield negative results (8). A notable staining pattern, designated as “neuropil pattern” observed in hippocampal and/or cerebellar tissue sections, typically indicates the presence of antibodies against neuronal surface antigens. Furthermore, studies have found that individual neuronal surface antigens have antigen-specific immunostaining patterns that can be used as biomarkers to estimate the target NS antigen (9). For example, the typical staining pattern for LGI1 shows intense staining in the dentate gyrus, middle of the dentate hilus molecular layer, and cerebellar molecular layer. This result was distinct from that observed for CASPR2, AMAPR, and GABA_B_R. However, the limitations of TBA include the possibility of false-negative results due to low antibody titers and the inherent difficulty in identifying novel antigens solely based on staining patterns. Consequently, the TBA results should be interpreted with caution when predicting the presence of NS-Ab or estimating the NS antigen.

ADAM23, a non-proteolytic member of the ADAM family, is primarily expressed in the hippocampus and cerebellum (10). As a presynaptic transmembrane protein, it modulates VGKC membrane expression and potassium currents, thus playing a critical role in neuronal excitability (11). In contrast, ADAM23 interacts with LGI1 to form a cross-synaptic complex, ADAM23-LGI1-LGI1-ADAM22, which plays a role in synaptic transmission (12). Studies have shown that the ADAM23 gene is a common risk locus for idiopathic epilepsy in dogs (13). ADAM23^−/−^ mice exhibit severe seizures and die by 2 weeks of age (14). Additionally, ADAM23 promotes neuronal differentiation, and its downregulation reduces neurite length and induces cell apoptosis (15). We speculate that the anti-ADAM23 antibody interferes with ADAM23 function, resulting in epilepsy and widespread neuronal dysfunction.

Given that ADAM23 is also a component of the VGKC complex, it may be important to screen for anti-ADAM23 antibodies in patients suspected of having AE. Muñoz-Sánchez et al. developed a method co-transfecting LGI1 and its natural synaptic ligand ADAM23 in HEK293 cells to enhance antibody detection sensitivity (16). However, while this approach demonstrates potential application value, it overlooks the possibility that ADAM23 may be an independent target antigen in AE, increasing the risk of misdiagnosis.

Antibody subclass analysis in this case revealed that the patient’s anti-ADAM23 antibodies were predominantly of the IgG4 subclass rather than the more commonly seen IgG1 subclass. IgG4 accounts for approximately 3%–5% of total IgG antibodies. IgG4 antibodies are unique because of their ability to undergo “Fab-arm exchange” and their inability to activate the complement system, which makes them immunomodulatory (17). Studies suggest that corticosteroids tend to exhibit better immunosuppressive effects than IVIg in various IgG4-related autoimmune diseases (IgG4-AID) (e.g., anti-LGI1 encephalitis) (18). This may be due to the fact that IVIg primarily exerts its effects through modulation of the complement pathway, while IgG4 antibodies themselves do not activate complement, thus limiting the role of IVIg in IgG4-AID. Consistent with this, our patient demonstrated a favorable response to corticosteroid treatment, which is likely linked to the characteristics of the dominant IgG4 subclass. Consequently, antibody subclass analysis may offer valuable guidance for tailoring appropriate therapeutic strategies. However, given that anti-ADAM23 antibody-associated encephalitis remains very rare, accumulating more real-world cases is essential to gain a deeper understanding and better treatment experience for IgG4-related autoimmune diseases.

In summary, the anti-ADAM23 antibody may be an overlooked autoantibody against the VGKC complex. To avoid underdiagnosis of anti-VGKC complex-associated AE, CBA detection of anti-ADAM23 antibodies may be essential. Further studies are required to elucidate the underlying pathogenesis and the effector mechanisms.

## Patient perspective

Understanding that her condition is quite rare, the patient’s son agreed to share this case with us.

## Data Availability

The original contributions presented in the study are included in the article/**Supplementary Material**. Further inquiries can be directed to the corresponding authors.
