# Supplementary material for: Case Report: Anti-ADAM23 antibody: an overlooked autoantibody against VGKC-complex in autoimmune encephalitis
Source: Front Immunol. 2025 May 30;16:1589360. doi: 10.3389/fimmu.2025.1589360 (PMC12162596; doi:10.3389/fimmu.2025.1589360)
Supplement: Supplementary file 1 [file Table1.docx]

Supplementary Material

**Supplementary Method 1** Cell-based indirect immunofluorescence assays（IIF-CBA）

The in-house cell-based indirect immunofluorescence assays were perfomed by Bejing Shenhaite BioTechnology company. Commercial plasmids containing full-length human ADAM23 cDNA(NM_003812) and full-length human LGI1 cDNA（NM_005097）were purchased from Vigenebio (Shangdong, China).For higher protein expression levels, the cDNA of ADAM23 and LGI1 were subcloned into the pcDNA3.1-3×Flag vector(Life Technologies, Invitrogen) individually.HEK293 cells were transiently transfected with the ADAM23 plasmid alone or cotransfected with the LGI1 and ADAM23 plasmids, and 24 hours later the cells were fixed with pre-chilled acetone.The cells obtained above were used as substrates for indirect immunofluorescence assay,serum samples were diluted at a 1:10 dilution using PBS-1% BSA（CSF samples need not to be diluted）, then incubated with pre-fixed transfected cells for 30 minutes. Immunodetection was performed using an goat anti-human IgG antibody conjugated with AF488 (109-545-003, Jackson ImmunoResearch Laboratories, USA) for 30 minutes.The results were observed under a fluorescence microscope.

**Supplementary Table1**Detection of anti-ADAM23 Antibodies in 9 Serum and 6 CSF Samples from Patients Previously Diagnosed With anti-LGI1 Encephalitis

| **Patient ID** | **Sample type** | **ADAM23+LGI1 HEK293 cells** | **ADAM23-only HEK293 cells** |
| --- | --- | --- | --- |
| **1** | **Serum** | **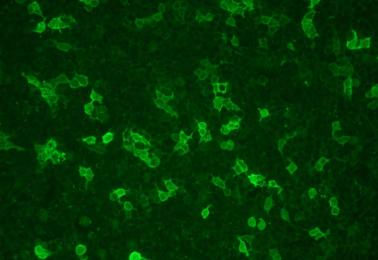** | **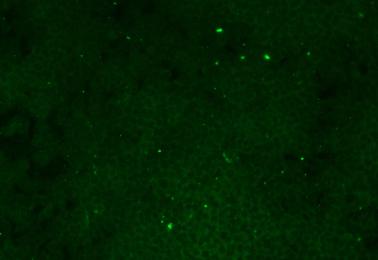** |
| **2** | **Serum** | **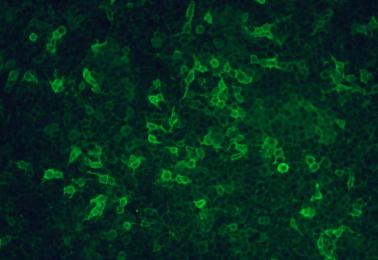** | **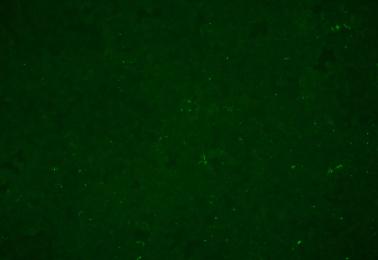** |
| **3** | **Serum** | **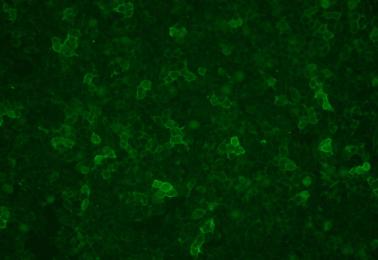** | **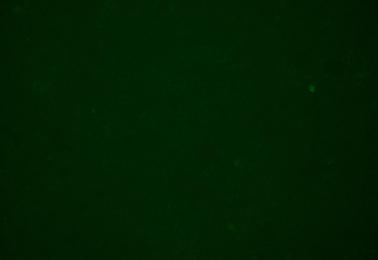** |
| **4** | **Serum** | **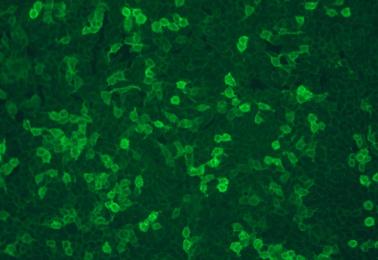** | **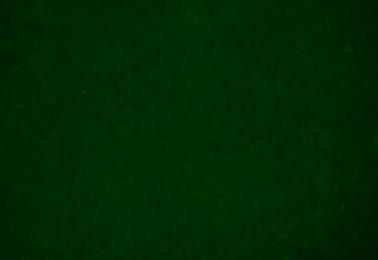** |
| **5** | **Serum** | **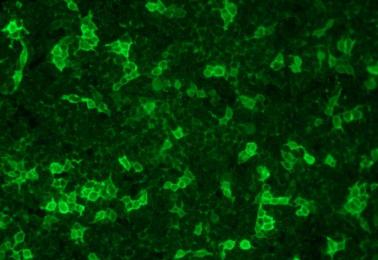** | **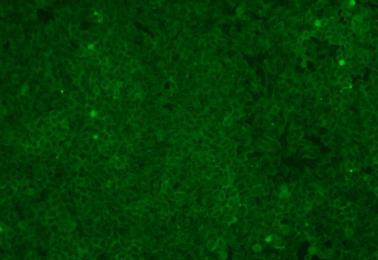** |
| **6** | **Serum** | **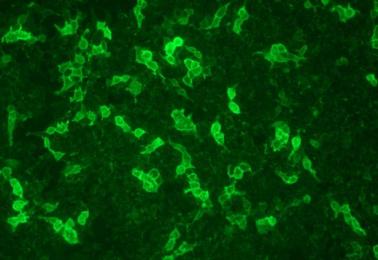** | **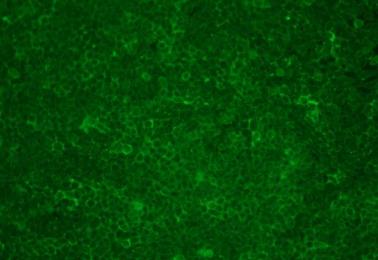** |
| **7** | **Serum** | **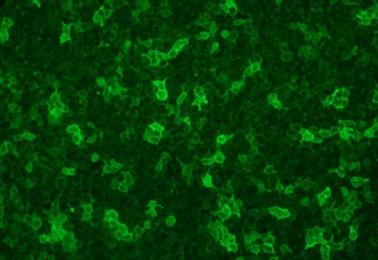** | **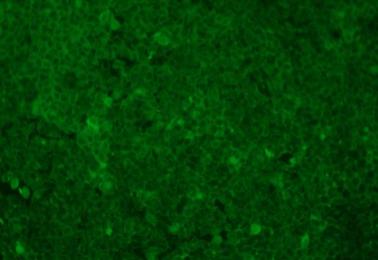** |
| **8** | **Serum** | **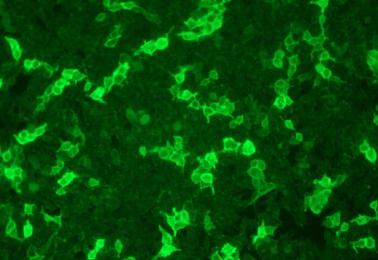** | **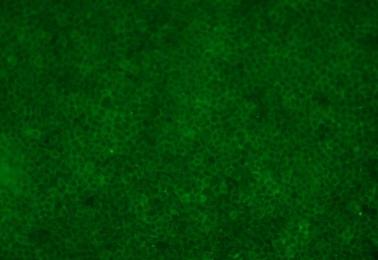** |
| **9** | **Serum** | **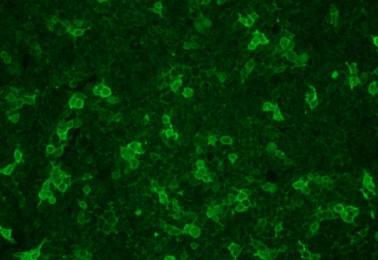** | **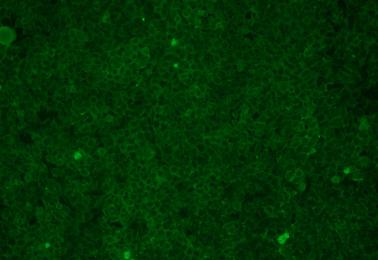** |
| **10** | **CSF** | **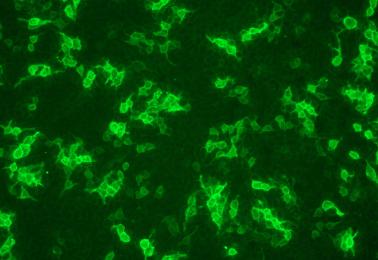** | **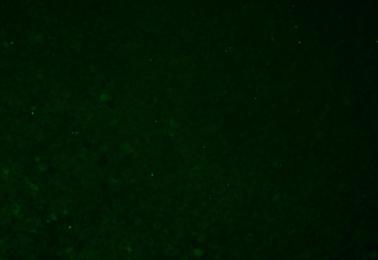** |
| **11** | **CSF** | **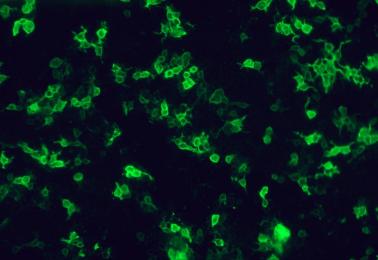** | **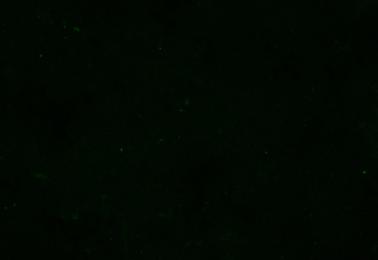** |
| **12** | **CSF** | **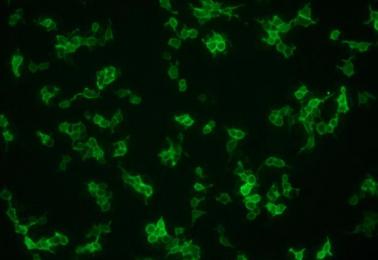** | **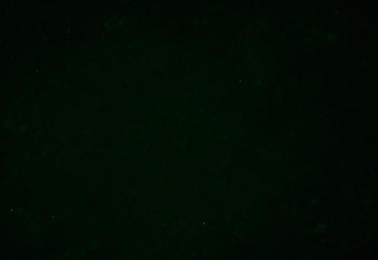** |
| **13** | **CSF** | **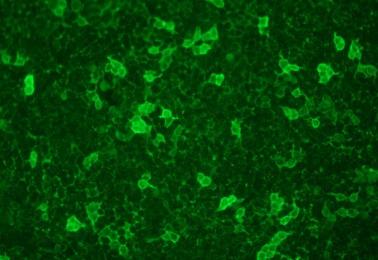** | **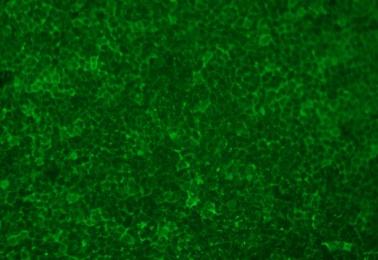** |
| **14** | **CSF** | **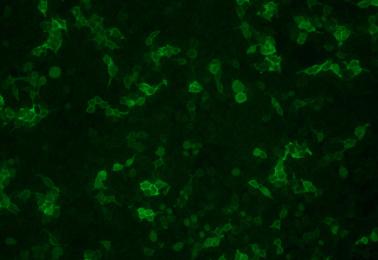** | **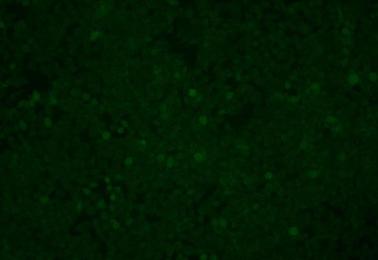** |
| **15** | **CSF** | **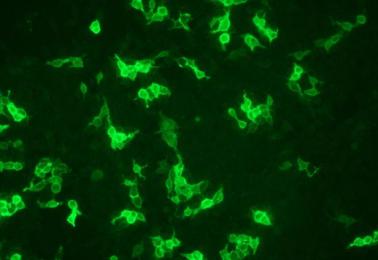** | **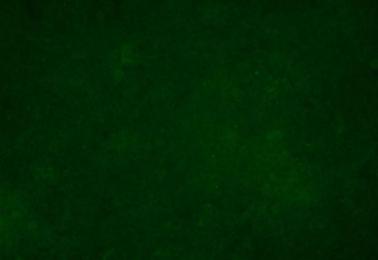** |
